# Strangers in a Strange Land: Designing a Mobile Application to Combat Loneliness and Isolation Among Foreign University Students

**DOI:** 10.1007/s41347-020-00171-6

**Published:** 2020-10-08

**Authors:** Rogério Augusto Bordini, Johann-Christoph Münscher, Kim Annabell Baumgartner, Sara Hagos, Jennifer Hornig, Stefano Gampe, Berkay Yaman, Oliver Korn, Philipp Yorck Herzberg

**Affiliations:** 1 Offenburg University, Offenburg, Baden-Württemberg Germany; 2 Helmut Schmidt University, Hamburg, Germany

## Objectives

A report from the World Economic Forum (2019) stated loneliness as the third most prevalent societal stressor in the world. This psychological strain, which was recently classified as harmful as smoking 15 cigarettes a day and more dangerous than obesity (CIGNA 2018), can also increase the risk of depression and other mental disorders due to the negative impacts on physical, mental, and social health (Cacioppo et al. 2002; Hawkley et al. 2003). Moreover, research shows that loneliness tends to be experienced more severely by young adults than other age groups (Rokach 2000). This is the case with students who face profound periods of loneliness when attending university in a new place (Diehl et al. 2018). A global survey conducted by Sodexo (2017) with 4027 students (aged from 18 to 25) from six countries (China, India, Italy, Spain, the UK, and the USA) reported that around a third of the participants (32%) said they experienced loneliness at university. A cross-national study with university students found associations between loneliness and subjective health status, such as sleeping problems, tobacco use, aggressive behavior, injury, and sexual risk behavior (Peltzer and Pengpid 2017). Furthermore, students have become even more affected by loneliness due to the absence of face-to-face contact caused by the social isolation measures adopted to reduce the COVID-19 spread (Elmer et al. 2020).

Besides facing stress factors in academic life such as teachers’ pressure for achieving good grades, acceptance difficulties in social circles, and the fear of professional failure, foreign students tend to face additional obstacles. They need to deal with a foreign language, finances, accommodation, day-to-day living problems, racial discrimination, personal autonomy, and the recreation of identity in a new setting, which can make them more susceptible to loneliness (Baker and Siryk 1986). Sawir et al. (2007) conducted a security investigation with 200 international students from more than 30 different nations in an Australian institution. The authors stated that international students were more likely to experience cultural loneliness, which is triggered by the absence of the preferred cultural and/or linguistic environment.

Sawir et al. (2007) and Vasileiou et al. (2018) suggested that creating stronger bonds among international and local students in the educational setting is a possible solution to a forward move on loneliness. Digital technologies have also been seen as promising interventions to address social isolation, loneliness, and mental health through social online environments (Shah et al. 2019) and mental health apps (MHapps) (Jansen-Kosterink et al. 2018). For instance, a number of institutions have been promoting apps to help manage students’ mental health as a possible solution to the universities’ budget limitations to offer more counseling services (Kern et al. 2018). Some potential benefits of using MHapps are as follows: portability, immediacy, and accessibility; support to rural populations; people on waiting lists for face-to-face services; difficult to engage groups; and affordability, convenience, and anonymity (Marshall et al. 2019).

Furthermore, initial studies suggest possible benefits of serious games and gamification for psychological and behavioral changes (Merry et al. 2012), as they might increase appealing, engaging, and effectiveness potentials of mental health interventions by offering immersive experiences to support behavioral modeling and social learning (Fleming et al. 2017).

Although these resources are seen as promising solutions, their efficacy to tackle students’ loneliness and social isolation remains contested due to the lack of evidence of the health benefits in integrating these technologies into university settings. In addition, there is little information on design recommendations for apps targeting this purpose. Therefore, it is from these assumptions that the project Noneliness, a gamified social mobile app, is being developed. It intends to provide a safe and collaborative social network for local and international students. Hence, in this study, we investigated participants’ perceptions, interpretations, and opinions concerning the design principles applied in the app’s first high-fidelity prototypes. Two evaluation phases with international students visiting a German university were employed, and the results are presented alongside the app’s design.

## Methods

The findings presented in this article were obtained through a preliminary exploratory study conducted at the Offenburg University as part of the Fighting Loneliness project promoted by the institution’s Affective & Cognitive Institute (ACI) from October 2019 to February 2020. The initiative’s main objective was to answer the research question “How should an app be designed to reduce loneliness and social isolation among university students?” with the collaboration of the institution’s students. In order to reach a possible answer, the ACI's team considered the following procedures:

- Literature review on loneliness intervention, mental health apps, and gamification for mental health research to identify the main solutions, challenges, and recommendations, as well as the related works.
- Critical analysis of the initial application design (version 1) and creation of a new improved user interface (UI) to be further explored with target audience in an A/B testing. It involved a set of activities developed in iterative cycles such as (i) brainstorm, (ii) research, (iii) preparation of scripts and documentation, and (iv) coding and testing.
- Design evaluations with the target audience through qualitative and quantitative research methods and successive refinements.

The study’s materials and data can be accessed at the Open Science Foundation platform (https://osf.io/jpsdb).

### App Design

The general concept behind Noneliness app, designed by the work’s first author, was derived from common UI and application design concepts with the specific goal of addressing loneliness among university students. Initially, a context analysis was carried out through semi-structured interviews with three psychologists and seven undergraduate students (Bordini 2018) at the University of Campinas. As students reported that many could not receive psychological care due to the high semestral demands of the university’s counseling service, these initial findings implied that a free mobile application could work as an accessible resource to motivate students to promote mutual aid. In addition, most participants stated dissatisfaction in seeking psychological support on social networks (e.g., Facebook) due to identity exposure and difficulties in finding local supporting groups.

**Fig. 1:**
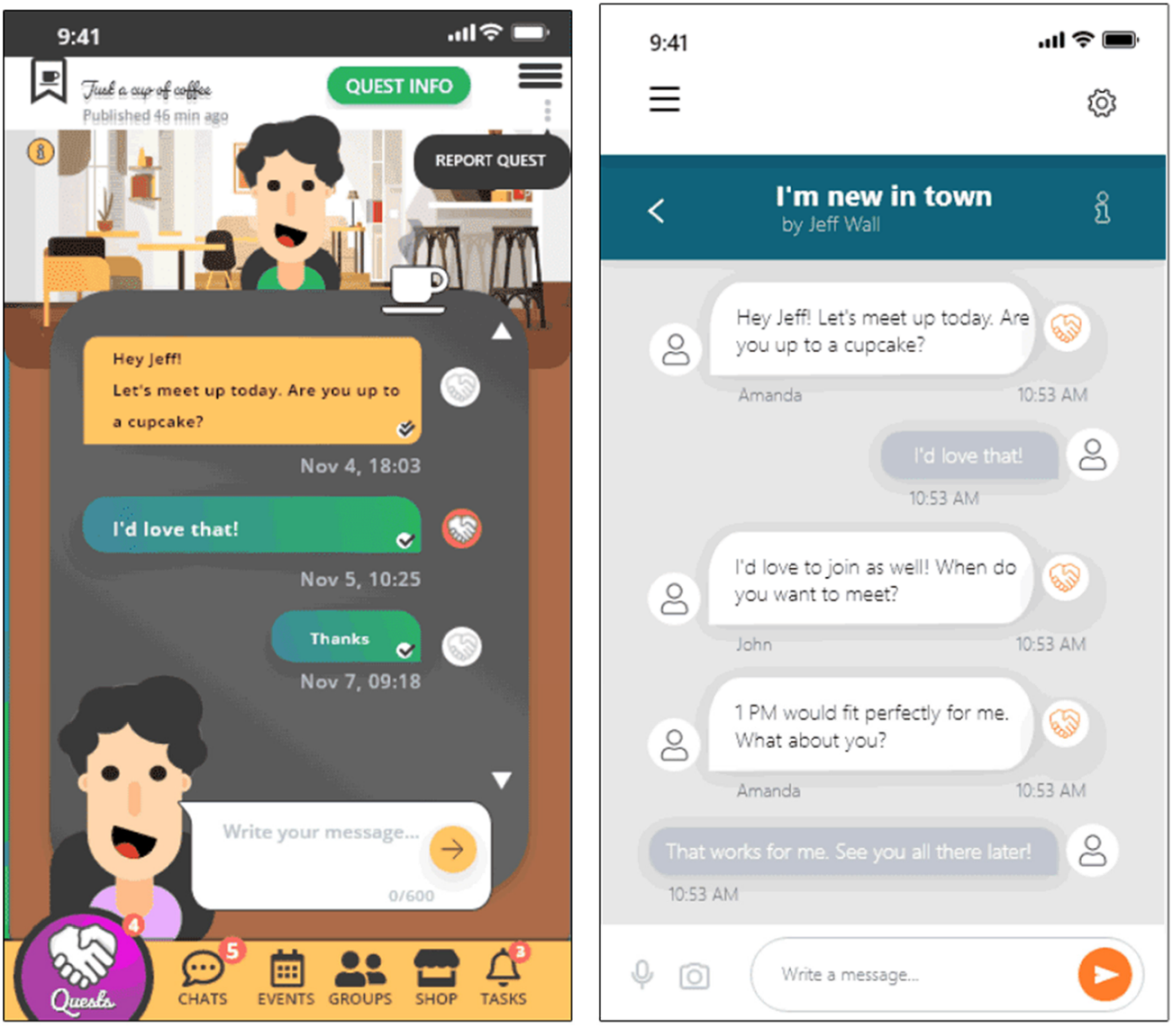
Noneliness app's Chat screen (version 1 on the left and version 2 on the right). Version 2 was the most preferred according to the survey data (52.88%)

After receiving positive feedback from the target audience through usability tests with the first prototype version in 2017 (Bordini 2018) (Fig. 1), the interface was redesigned in the beginning of the Fighting Loneliness project in order to meet the state-of-the-art on designing high-efficacy mental health mobile apps (Bakker et al. 2016; Chandrashekar 2018) and loneliness reduction interventions through digital technology (Shah et al. 2019) (version 2):

Based on a set of recommendations on gamification in mental health (Fleming et al. 2017), we adopted game elements to investigate whether these features could improve users’ engagement within the application through rewards, points, and level progression.

The app’s initial design considered the following features:

- Quests: message feed where all users’ posts are displayed as “quests” through which local users can find emotional support, invite for meet ups, or ask favors.
- Avatars: as participants in the first study (Bordini 2018) stated discomfort exposing themselves to talk about personal problems online, customizable 2D avatars were thought of as a way to encourage reclusive users for app usage.
- Events: allow users to join or create a local event for a certain activity at a specified time (e.g., art exhibition, concert, and meet up). These events are pulled by the users themselves and can also be used by institutions to create and promote their own social and institutional activities.
- Chats: whenever a Quest or Event is accessed, a text-based chat window opens. It allows users to talk about the proposed subject and exchange *Gratitude Points* by tapping a button displayed next to each message (Fig. 1). These points are used solely to demonstrate appreciation to users and to purchase new avatar items in the application’s Shop option (explained below).

The app also has sub-functionalities such as *Shop* for acquiring avatars accessories in exchange of *Gratitude Points* (no real money is used) and *Profile* where personal information and interests are displayed.

These app’s main features were also designed to be easily adopted in a variety of scenarios, so both students and university advisors could use them according to their needs. Some possible uses by institutions are as follows: setting up support groups for students who are facing various difficulties in their academic life (e.g., study problems, vocational guidance), create welcome events, and raise awareness on psychological support (crisis lines, counseling centers, access to experts, etc.). This would narrow the existing gap between the institution’s administrators and students, as the latter often do not know how to proceed in the face of an emotional and psychological difficulty or are unable to access such services due to the long queues to receive appropriate care.

### Procedure

Two user-experience attitudinal assessments were conducted. Letters were sent to the Offenburg University’s mailing list inviting international students for both study phases, which happened within a 1-month interval. Both interventions took place at the same institution and were based on:

I. Focus group discussion (FGD) through a safe and informal environment where participants could spontaneously express their reactions, beliefs, and ideas.
II. Paper-based and online survey consisted of 56 questions. Besides gathering sociodemographic data (age, gender, country of origin), the questions were designed to capture the participants’ current situation (start and duration of stay in Offenburg), their social interactions, as well as attitudes towards social media and smartphone apps that employ gamification principles. Most questions employed five-point Likert scales, some allowed freeform answers, and other provided multiple choices.

The Likert scale response format was used in the survey to calculate scores organized in aspects relevant to the current research question (Table 1). Relations between these scores were explored to identify relevant aspects with regard to the app design.

**Table 1:** Survey items

Aspects	No. of items	Description
Personal information	5	Information about the participants’ profile, age, gender, native countries, and current situation at the Offenburg University
Perception of culture	9	Perception of how welcoming and approachable the foreign culture is perceived. This perception is dependent on the actual culture present in society but is also greatly impacted by an individual’s approach to the foreign culture. Feelings of loneliness in a foreign country can largely be attributed to a perceived disconnect between oneself and the surrounding culture (Rokach et al. 2001)
Socializing	9	Personal need and willingness to engage in social activities such as meeting people, friendly interactions, and asking for help.
Reclusion	9	Somewhat opposed to the socializing aspect yet independent, it describes the need to be alone from time to time and solving problems in isolation
Social media use	11	General view of social media services as a viable tool in social interactions
Interest in gamified apps	5	Overall interest and openness to try and use apps that employ gamification principles
Noneliness *app* impressions	8	Participants’ perception on the app’s general concept (visual identity, UIs, features) and comparison between the two designed interfaces (Fig. 1)

As until the study period, it was not possible to find a reliable instrument capable of covering all the raised aspects necessary to obtain relevant data for the app’s future development (e.g., intersection between culture, social interaction, technology, and gamification), and the questionnaire’s items were derived from different quantitative and qualitative studies which brought important findings concerning these topics.

Items related to perception of culture aspect were based on Sawir et al. (2007) questions’ guide used for interviewing international students. These questions covered a range of areas touching on the social and economic security of these students and their culture’s perception. Socializing and reclusion items were based on the UCLA Loneliness Scale (LS) (Russell 1996), a 20-item instrument designed to measure one’s subjective feelings of loneliness and social isolation. It focuses on participants’ evaluations of different qualitative aspects of their social networks and interactions. Social media use questions were elaborated based on Kim et al. (2016) who examined how college students’ psychological need to belong is associated with their use of social media and smartphones. The authors measured participants’ social media use and social engagement by asking how frequently they used to use social network sites for social activities. Questions about interest in gamified apps were inherent to the previous aspect, as a form to understand at what extent gamification is important to improve subjects’ engagement within a given digital resource.

As the Offenbug University administration had informed us that all international students had sufficient command of English in common, this language was chosen both for the creation of the app wireframes and for communication during data collection procedures.

## Results

FGD subjects, three females between 21 and 24 years old, from Mexico, India, and Brazil, were interviewed. Guided interviews were conducted, focusing on how participants experience living in a foreign country, their ways to make friends, use of technologies to reduce loneliness, and their impressions on the app’s design and UIs (A/B test). The app was demonstrated using interactive Adobe XD’s wireframes. Data analysis was based both on the notes and audio recordings taken during the session, identifying the participants’ recurring main ideas and criticisms about the discussed topic.

Participants mentioned that Offenbug University’s concept could approximate real and online users through some functionalities. *Events* was chosen as the best feature to help newcomer students find social opportunities. *Chats* and *Profile* were considered equally important for users’ identification and interaction. *Quests* was considered less effective in promoting real-world meetings, but it could help lonely people who do not have someone to relate to. As stated by one participant “[...] it is important for students to have someone to share and also find people that relate to you [...]”. *Avatars* and *Shop* were the least important for them, as students focused more on the app’s social aspect rather than customizing avatars. Concerning the app’s UI, the subjects stated version 1 (Fig. 1) as “overloaded with illegible fonts and confusing colors”. Version 2 was more accepted as “simpler, targeted and clearly arranged”.

In the survey, participants were *N* = 30 (Table 2), and the correlations between the aforementioned aspects (Table 1) were calculated and are displayed in Table 3; absolute correlations ranged from *r* = .03 to .49. Datasets and scripts can be accessed in the Open Science Foundation repository.

**Table 2:** Demographic characteristics of survey participants

Demographic	Value	Frequency	%
Gender	Male	17	56.66
	Female	13	43.33
Age	20–21	2	6.25
	22–23	6	18.75
	24–25	5	15.63
	25+	17	56.66
Location	Asia	24	80
	Europe	2	6.6
	South America	1	3.3
	North Africa	1	3.3
	Central America	2	6.6

**Table 3:** Correlations among survey aspects

	Gender^b^	Aspects^a^					*M*/*SD*
		PC	S	R	SM	GA	
PC	− 0.12	*(0.52)*					10.13/2.1
S	− 0.04	0.26	*(0.77)*				29.60/5.9
R	0.23	− 0.29	− 0.5	*(0.71)*			25.53/5.88
SM	0.04	− 0.18	0.03	0.16	*(0.62)*		10.33/3.0
GA	0.02	− 0.08	0.36	− 0.18	0.15	*(0.62)*	12.53/3.0

Diagonals report internal consistencies of the scales (Cronbach’s α)

^a^*PC* perception of culture, *S* socialization, *R* reclusion, *SM* social media, *GA* gamified apps

^b^Point-biserial correlation (female = 1, male = 2)

Some survey respondents, *N* = 19, also answered a freeform question about what features they expected to find in such an app, ordered by the number of occurrences: receive relevant answers (26.31%); events updates (15.78%); accessibility and user friendliness (15.78%); safety and data privacy (15.78%); large community of users (15.78%); display users’ interests (5.26%); and anonymity (5.26%). Avatar usage was rejected by 74.07% of all respondents as they stated that they could not trust an account without the owner’s real photo.

## Conclusions

The multimethod approach used in this study helped us to collect potential evidence on how an app should be designed to reduce loneliness and social isolation among university students. The findings suggest that the application’s general concept is promising for the creation of stronger bonds among international and local students (Sawir et al. 2007).

FGD participants revealed their struggle to connect with other local and international students after their arrival due to the lack of social opportunities at the university. This suggests the need for institutions to create greater integration opportunities for local and newcomer students, such as social activities and psycho-education sessions to increase students’ sense of belonging (Vasileiou et al. 2018). The app, therefore, could contribute to the dissemination of such information and events (Jansen-Kosterink et al. 2018; Chandrashekar 2018; Bakker et al. 2016).

Evaluating the five scores calculated for the survey sample of students gives some insight into the special requirements and challenges for app design in this case. Unsurprisingly, socializing and reclusion are negatively correlated. Additionally, both scores show moderate correlation with the perception of culture with opposed directions. Individuals with higher expressions of socializing also report a friendlier perception of the surrounding foreign culture. Individuals with a greater tendency to isolate themselves report a less welcoming culture. This combination of self-isolation and negative perception of the surrounding context likely contributes greatly to feelings of loneliness and their negative impacts (Rokach et al. 2001). Critically, a clear difference between men and women can be observed in regard to socializing and reclusion. Men seem to have a more pronounced need to isolate themselves; men and women do not differ in their tendencies to socialize in this context. Therefore, the following conclusions for design considerations can be drawn. Gender and social media use have no to little connection to the willingness to try gamified apps. A moderate connection can however be observed with socializing. Additionally, reclusion exhibits a small negative correlation. This pattern may indicate a special challenge for the design process as individuals who would benefit the most show less willingness to try a gamified app. At the same time, those with tendencies to socialize will likely engage with such an app.

According to the results of both evaluations, features such as *Events* to increase social opportunities creation and the possibility of building an online supporting community through a functional and secure application were participants’ most expected functionalities. Although *Avatars* and gamification have been less accepted, both features will be maintained in the next version to better assess whether these elements can positively impact the resource usage. We will also adopt a more minimalist interface, as preferred by most participants in both studies (Fig. 1).

Currently an *alpha* app version is being implemented considering the collected results, and it will be released in the second semester of 2020. A Offenburg University full stack developer is developing the application using MySQL open source for database storage, PHP to manage its operations and HTML, and CSS and JavaScript for the front-end. For the next version, we are considering a semi-anonymous approach to attend both individuals with tendencies for reclusion and socializing according to the survey’s results, providing anonymity to those who just want to chat online and offer security to those who are looking for real-life interactions. Accessibility features (e.g., text-to-speech), geo-location optional functionalities (GPS) to facilitate *Events* search, and additional language options (German, Spanish and Mandarin) are also planned.

Given the study’s small sample size with self-selected participants and no testing with a functional app, further investigations can be focused on a randomized controlled trial in the second half of 2020, with the Offenburg University’s counseling service collaboration, as many students are experiencing social isolation due to the COVID-19 safety measures, a larger group of interested participants is expected. They can be divided into experimental and control groups to investigate how they can tackle loneliness by using Noneliness *alpha* app (experimental) and Facebook (control) for the same amount of time (about 3 months). Semi-structured interviews about the usability, experience, and their emotional states before, during, and after the contact with each app are expected to be taken alongside the UCLA-LS (Russell 1996) utilization to measure and compare loneliness and social isolation reduction with both apps.

The development of this application intends to evaluate, in a first moment, its effectiveness in a small university setting. However, in the future, depending on the resource acceptance and the results collected, it is expected that this application will be expanded and offered to other university communities around the world.
